# Relationship between corrosion and nanoscale friction on a metallic glass

**DOI:** 10.3762/bjnano.13.18

**Published:** 2022-02-18

**Authors:** Haoran Ma, Roland Bennewitz

**Affiliations:** 1 INM – Leibniz Institute for New Materials, Saarbrücken, Germany; 2 Department of Materials Science and Engineering, Saarland University, Saarbrücken, Germany; 3 Department of Physics, Saarland University, Saarbrücken, Germany

**Keywords:** atomic force microscopy (AFM), corrosion, friction, metallic glass, passive film

## Abstract

Metallic glasses are promising materials for microdevices, although corrosion and friction limit their effectiveness and durability. We investigated nanoscale friction on a metallic glass in corrosive solutions after different periods of immersion time using atomic force microscopy to elucidate the influence of corrosion on nanoscale friction. The evolution of friction upon repeated scanning cycles on the corroded surfaces reveals a bilayer surface oxide film, of which the outer layer is removed by the scanning tip. The measurement of friction and adhesion allows one to compare the physicochemical processes of surface dissolution at the interface of the two layers. The findings contribute to the understanding of mechanical contacts with metallic glasses under corrosive conditions by exploring the interrelation of microscopic corrosion mechanisms and nanoscale friction.

## Introduction

Metallic glasses (MGs) exhibit excellent mechanical properties including extraordinary hardness and strength [1–2]. Thus, MGs have emerged as novel wear-resistant materials with high potential in tribological applications [3–8]. Tao et al. [3] found that Zr-based MGs present a much smaller friction coefficient than other metals under dry-sliding conditions. W-based MGs were developed whose wear resistance was demonstrated to be comparable to classical tribological ceramics [6]. Hofmann et al. [7] reported that the wear resistance of CuZr-based MG gears is superior to that of high-performance steel.

Metallic glasses can be formed thermoplastically in the supercooled liquid regime [9–10]. This process allows for the application of MGs in microelectromechanical systems (MEMS) [11]. The tribological performance on the nanoscale is crucially important. Microscale bearings made of Ni-based MGs lasted four times longer than those machined from sintered alloy [12].

Corrosive degradation, as one of the major failure mechanisms of metals and alloys, is an important issue in engineering applications of MGs. Protective oxide films form on most metal surfaces and act as a barrier to the corrosive environment, thus impeding further corrosion. The corrosion properties of MGs, for example, the ability to passivate and to remain in the passive state in corrosive aqueous solutions, have been addressed in many studies using electrochemical methods, often combined with surface analytical techniques [13–15]. Wang et al. [13] reported that the passive oxide films are grown as a double layer structure on MGs with a corrosion product layer underlying an inner barrier layer in NaCl and Na_2_SO_4_ solutions. Since most metals and alloys are susceptible to corrosion when exposed to environmental conditions, the role of surface chemistry for friction must be investigated. At the macroscale, the existence of metal oxide surface films on MGs enhanced the wear resistance in corrosive solutions and the fluid lubricating films formed by solution and corrosion products on the surfaces reduced the friction coefficient [16–17]. The native oxide layers grown in the air were found to strengthen the friction coefficient and the wear resistance of MGs at the nanoscale [18–19]. The thermal oxidation caused a higher contribution of shearing and a significantly lower contribution of plowing to nanoscale friction and wear [20]. As far as we know, the effect of oxide films on the nanotribological properties of MGs formed in corrosive solutions has not yet been investigated, although it is important for miniaturized applications of MGs under corrosion conditions.

Recently, we investigated nanoscale friction on a Zr_63_Ni_22_Ti_15_ (ZrNiTi) MG in phosphate buffer after electrochemical polarization [21]. Our results demonstrated a new method to investigate in situ the structure of surface oxide films grown upon polarization in aqueous solutions using friction force microscopy. Here, we apply the same method to investigate differences in corrosion of ZrNiTi MGs after different periods of immersion time between two different solutions. On the one hand, the influence of corrosion on nanoscale friction on MGs is evaluated. On the other hand, nanotribological in situ experiments are implemented to reveal microscopic corrosion processes.

## Results and Discussion

### Potentiodynamic polarisation tests

**Figure 1:**
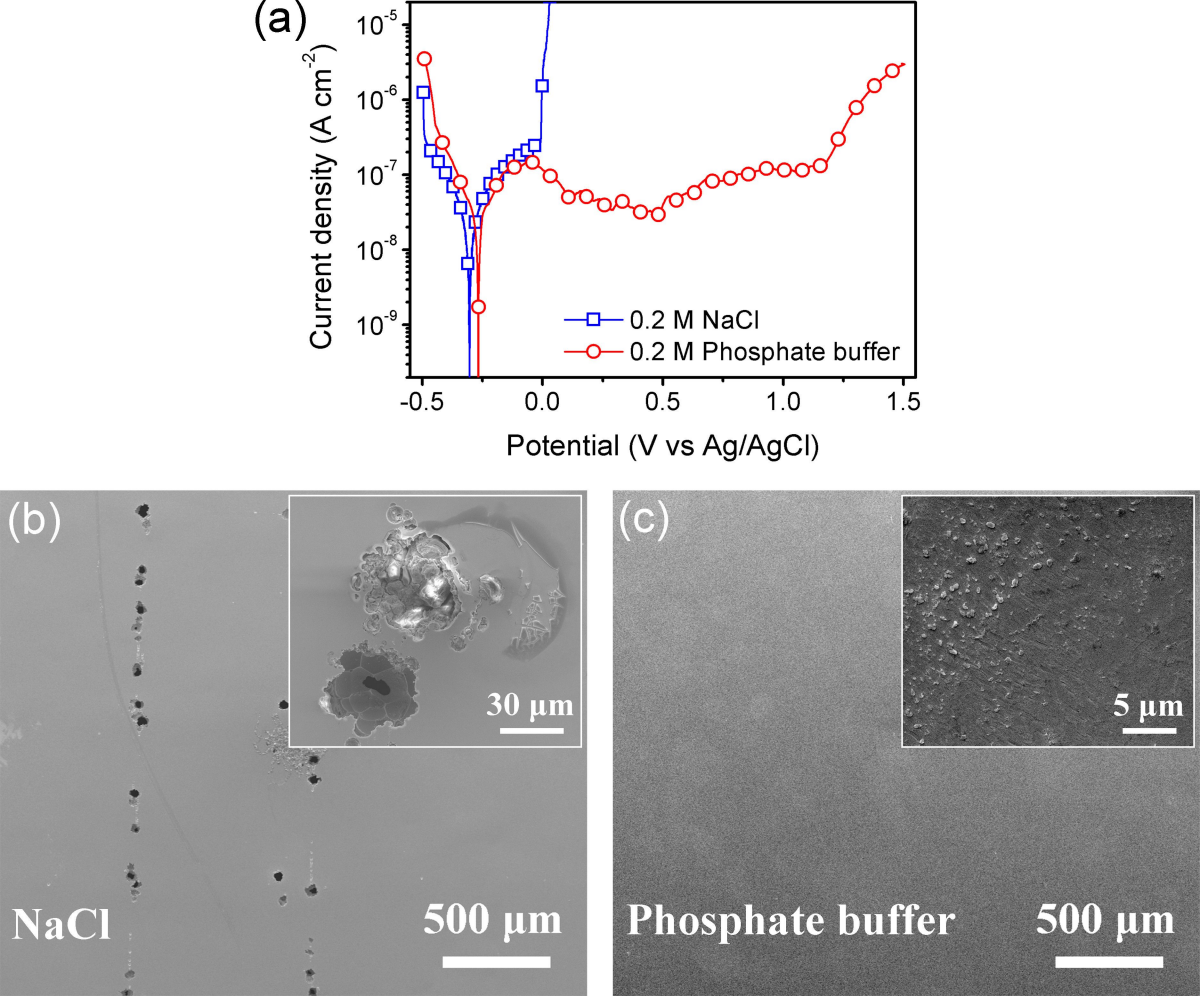
(a) Potentiodynamic polarization curves of Zr_63_Ni_22_Ti_15_ metallic glass in 0.2 M NaCl solution and 0.2 M phosphate buffer recorded in an electrochemical AFM cell. SEM images of the corroded surface after potentiodynamic polarization test in (b) NaCl solution and (c) phosphate buffer.

Phosphate buffer and NaCl solution were selected as test solutions because of their differences in corrosion of ZrNiTi MGs. Figure 1a shows potentiodynamic polarization curves of ZrNiTi MGs in NaCl solution and phosphate buffer recorded in an electrochemical AFM cell. In NaCl solution, no passivity is observed during anodic polarization. The current density increases rapidly even at a low applied potential (approx. 0 V). In contrast, the ZrNiTi MG in phosphate buffer is passivated spontaneously with a wide passivation region (−0.05 to 1.2 V). These results indicate a significantly higher corrosion resistance of the MG in phosphate buffer compared to NaCl solution.

After potentiodynamic polarization, the corroded surfaces of ZrNiTi MGs were investigated by scanning electron microscopy (SEM); the images are shown in Figure 1b,c. Corrosion pits with a lateral extension of tens of micrometers were observed on the surface polarized in NaCl solution, indicating that the chloride-containing solution initiates localized pitting. The inset in Figure 1b shows the magnified image of typical corrosion pits. No such pits are found after polarization in phosphate buffer (Figure 1c). The surface is mostly smooth and only some parts exhibit signs of increased roughness (inset in Figure 1c). We conclude that the polarization-induced surface modifications proceed uniformly in phosphate buffer.

Pitting has been reported for many MG surfaces after polarization in chloride solutions [22–25]. Pitting corrosion is induced by heterogeneity or discontinuity of the amorphous matrix, for example, by crystalline inclusions [24]. On the surface shown in Figure 1b, pitting is always distributed along a line. Wang et al. [26] found that pitting occurs preferentially at the shear offsets on a pre-deformed Zr-based MG due to the higher chemical activity of offset sites compared with the surrounding flat region. This influence of surface morphology was also shown for copper surfaces, where it was suggested that more electrons escape in the vicinity of a peak than in a valley [27]. A surface undulation with parallel valleys on our ZrNiTi MG ribbons may be the reason for the distribution of pits along lines. Another possible reason is residual stress, indicated by the strip curled state of MG ribbons after preparation. In contrast, the ZrNiTi MG is not susceptible to pitting corrosion in phosphate buffer. Phosphates are generally used as effective inhibitors to minimize the risk of rebar corrosion [28–29]. The phosphate ions hinder the initiation of pitting by their buffering capacity, which impedes acidification inside the pits and promotes the repassivation of initially metastable pits [28]. The stability and protection effect of the surface film is also improved when phosphates are involved in the film formation [29].

### Nanoscale friction after immersion

**Figure 2:**
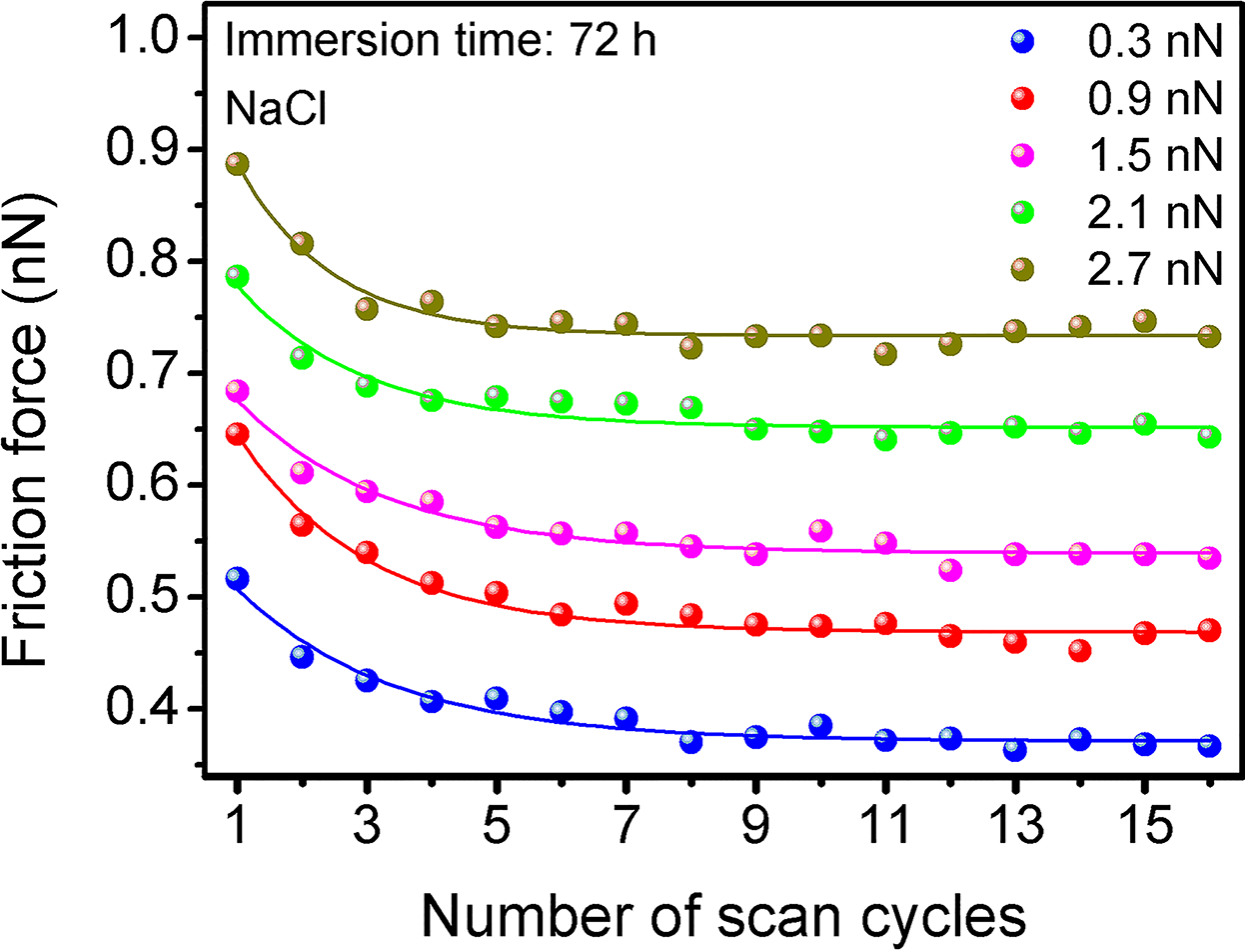
Friction force as a function of number of scan cycles on Zr_63_Ni_22_Ti_15_ metallic glass after immersion into 0.2 M NaCl solution for 72 h. The smooth curves are fits of the data to an exponential decay function.

The development of friction force with the number of scan cycles after immersion in NaCl solution for 72 h is shown in Figure 2 for experiments at different applied loads. Please note that all friction experiments are performed in the immersion solution without applying a potential. The friction force initially decreases with the number of scan cycles and then reaches a steady value at all loads. This decay of friction is also found for all other parameters, that is, after immersion in NaCl solution for 1 and 24 h, and in phosphate buffer for all periods of immersion time. Different from the polarization result (Figure 1b), there are no pits on the sample surface even after immersion in NaCl solution for 72 h. This weak corrosion during immersion without applied potential will be discussed in more detail below.

**Figure 3:**
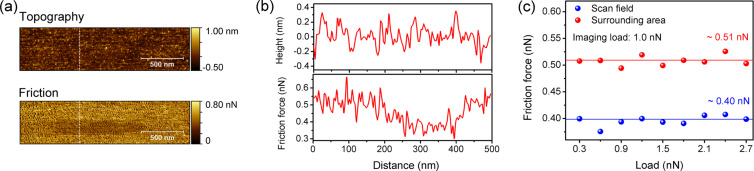
(a) AFM topography and friction force images recorded on Zr_63_Ni_22_Ti_15_ metallic glass after immersion into 0.2 M NaCl solution for 72 h and after scanning the central area (1.0 × 0.125 μm^2^) 16 times at a normal load of 1.5 nN (imaging load: 1.0 nN); (b) cross section of the topography and friction image corresponding to the lines drawn in (a); (c) friction force of the scan field and surrounding area as a function of the normal load applied during 16 repetitive scans in the scan field. Friction data were extracted from images recorded at an imaging load of 1.0 nN.

Figure 3a shows the topography of the scan field and corresponding friction force images after 16 scan cycles in the central 1.0 × 0.125 μm^2^ region at an applied normal load of 1.5 nN. The images of the scan fields in this work have all been recorded during the first scan in the surrounding area after friction tests of 16 scan cycles in the scan field. There is no measurable height difference between the central repetitively scanned field and the surrounding area in the topography image. However, we do observe a contrast between these two areas in the friction force image, revealing the position of the scan field. The corresponding line-scan profiles across the scan field and surrounding area are presented in Figure 3b. The friction force is significantly smaller on the scan field, while the height of the scan field and surrounding area do not differ.

In Figure 3c, the friction force on the scan field after 16 repetitive scans and on the surrounding area are plotted for the different normal loads that were applied during the repetitive scans within the field. The friction values were calculated from images like the one shown in Figure 3a. Please note that each scan field was produced with the respective load on a different surface area. The friction force of the surrounding area is constant about 0.51 nN. This is expected because the imaging load is constant and the area surrounding the scan fields is not altered by preceding scans. The friction forces of the scan fields produced at different normal loads are also similar, but about 0.40 nN lower than that on the surrounding area. This observation lets us conclude that the tip slides on a surface that has the same characteristics after repetitive scanning at different loads.

Surface oxide films formed during corrosion have been reported to exhibit a double layer structure with a dense, protective inner layer and a porous, precipitated outer layer [18,21,30–32]. The outer layer originates from the dissolution of the underlying dense layer and the MG substrate. The characteristics of the friction results reported in Figure 2 and Figure 3 reflect the double layer structure of surface oxide films. A similar correspondence between friction and topography, on the one hand, and double layer structure, on the other hand, was observed for MG surfaces after polarization in phosphate buffer [21].

We will now discuss our experimental results in view of the double layer structure of the surface oxide film formed during immersion. The tip penetrates the outer layer and slides on the surface of the inner layer beginning with the first scan cycle. The friction force in the first scan cycle is then the sum of two contributions. The first contribution is the friction force of the tip sliding on the surface of the inner layer (friction force of the inner layer). This contribution is quantified as the steady value of friction force in data fits of Figure 2. The second contribution is the plowing force needed to remove the outer layer in front of the tip (friction force of the outer layer). This contribution is quantified as the difference in friction force between initial and steady value in data fits of Figure 2. During repeated scanning, the gradual removal of the outer layer by the action of the sliding tip leads to the decrease of friction, and the friction of the outer layer eventually reaches zero. A detailed analysis of this process can be found in [21]. The lack of height contrast in Figure 3a is explained by penetration of the AFM tip into the soft outer layer surrounding the scan field. No height difference can be measured between the surrounding area, where the tip penetrates the outer layer, and the scan field, where the outer layer was removed. Friction, however, is higher in the surrounding area, where the tip is still plowing the outer layer. Zhao et al. [33] reported a friction decay with repeated scanning on a graphene-coated Cu substrate, caused by the hardening of the underlying Cu substrate. For the oxidized metallic glasses and the scanning conditions investigated here, no friction mechanism that is due to the plastic deformation was observed (see Supporting Information File 1 for full experimental data).

**Figure 4:**
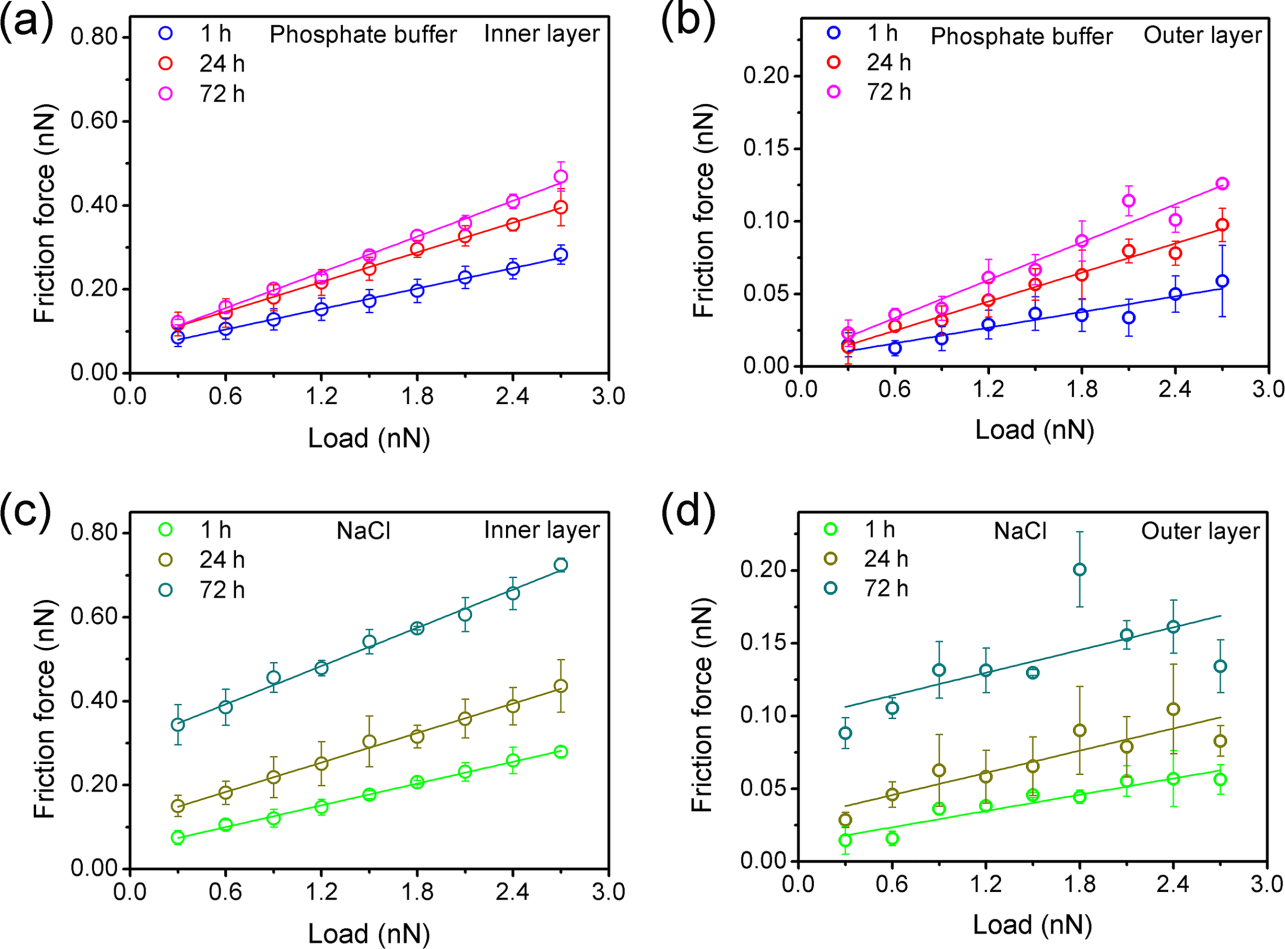
The dependence on the applied normal load during the repetitive scans of: (a) friction force of the inner layer and (b) friction force of the outer layer in phosphate buffer; (c) friction force of the inner layer and (d) friction force of the outer layer in NaCl solution. Solid lines are linear fits. Each data point is the average value of three replica experiments and error bars represent the standard deviation.

The friction forces are compared for the inner and outer layer in Figure 4 for the different normal loads applied during respective scanning. For both solutions and all immersion times, the friction force of the inner layer increases linearly with the normal load (Figure 4a,c). Adhesion contributes significantly to the friction force, that is, a friction force is measured even at zero externally applied load. A linear increase of the friction force with the applied load is also observed for the outer layer after immersion in phosphate buffer (Figure 4b). We attribute the increase in friction for the outer layer to the contact area between the outer layer and tip, which grows in parallel to the increased contact area of the inner layer and tip apex at a higher normal load [34–35]. The friction force of the outer layer reveals the lateral plowing resistance of the outer layer to the sliding tip, which must depend on the shear strength of the layer and its structure. The friction data for each respective load is recorded on a different spot of the surface. In phosphate buffer, the perfect regularity of the linear dependence of friction on load in the outer layer indicates a similar plowing resistance in different spots and, thus, a laterally uniform outer layer. The dissolution process is uniform on the surface in phosphate buffer, even after immersion for 72 h. This is not the case for the NaCl solution (Figure 4d), where a significant scattering of friction values is observed, especially after a longer immersion. The general trend is still towards higher friction forces of the outer layer for the increasing load. The outer layer formed during immersion in NaCl solution is non-uniform, indicating an inhomogeneous dissolution process on the surface. This difference in corrosion processes between phosphate buffer and NaCl solution agrees well with the results of potentiodynamic polarization (Figure 1), although the inhomogeneity of dissolution is still not sufficient to induce pits on the sample surface after immersion in NaCl solution.

### Relationship between corrosion and nanoscale friction

**Figure 5:**
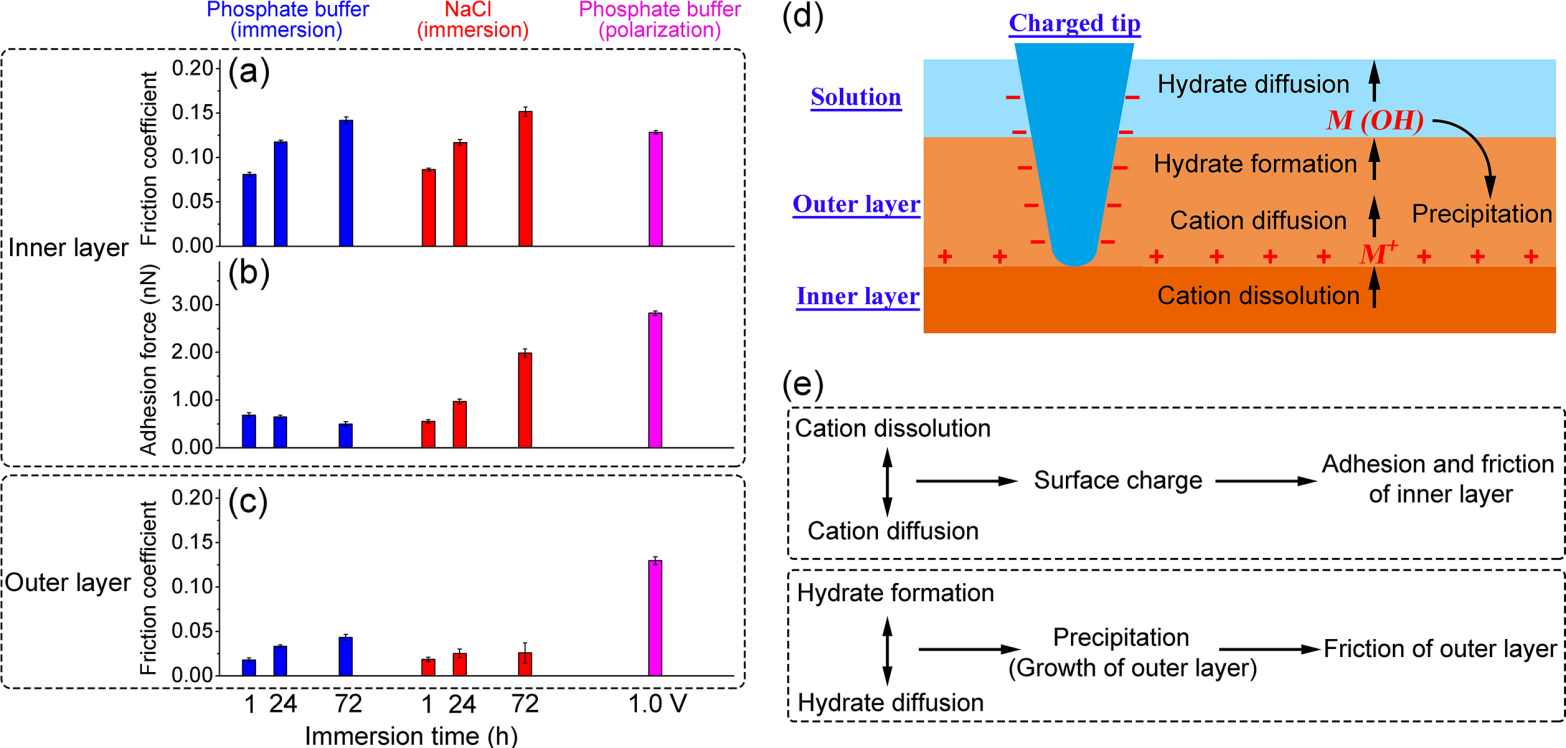
The dependence on immersion time of: (a) friction coefficient of the inner layer; (b) adhesion force of the inner layer; (c) friction coefficient of the outer layer. Data are obtained from linear fits in Figure 4 and error bars represent the errors in the fits. Data for corroded surfaces after polarization in phosphate buffer for 80 min at 1.0 V vs Ag/AgCl are shown for comparison. (d) Schematic illustration of physicochemical processes at the interfaces of the surface oxide film related to the surface dissolution during corrosion. M*^+^* represents dissolved metal cations, M(OH) denotes hydrates formed by reactions of metal cations with the solution. (e) Summary of the influence of corrosion on friction and adhesion of the inner layer and friction of the outer layer.

We will now compare the friction results in phosphate buffer and NaCl solution. Figure 5a–c displays the dependence of the friction coefficient and the adhesion force on immersion time for inner and outer layers. The friction coefficient is calculated as the slope of a linear fit to the friction force versus normal load data (Figure 4). The adhesion force of the inner layer versus the AFM tip is determined as the abscissa intercept of the linear fit at the zero friction force (Figure 4a,c). Data of the corroded surface in phosphate buffer after polarization for 80 min at 1.0 V vs Ag/AgCl [21] are shown for comparison. When a potential is applied in NaCl solution, the solution turns cloudy after a few minutes with a large amount of corrosion products released into the solution, suggesting a serious corrosion. This degradation impedes AFM friction experiments that are based on optical detection through the solution.

After immersion for the same time in phosphate buffer and NaCl solution, the friction coefficients of the inner layer are equal within error. After immersion for 72 h, they become comparable with the friction coefficient after polarization in phosphate buffer (Figure 5a). Passivation is a kinetic process in which growth and dissolution of oxide films occur simultaneously [30]. Consequently, the inner layers in these three cases can be expected to be different in structure and composition, in view of the different dissolution processes, which will be discussed in detail below. It is therefore important to note that the frictional response of the inner layer develops similarly during immersion in NaCl and phosphate buffer and that the friction coefficient of the inner layer is similar after long immersion and after polarization in phosphate buffer.

The adhesion force remains constant with increasing immersion time in phosphate buffer and is much smaller than after polarization (Figure 5b). The anodic polarization results in a net positive surface charge [36–37], caused by the accumulation of the dissolved metal cations on the inner layer and strengthens the adhesion of the negatively charged silicon AFM tip [38–39]. Figure 5d depicts schematically this charge buildup at the surface of the inner layer, which involves two physicochemical processes. Metal cations are generated at the interface between the inner and outer layer by the dissolution of the inner layer oxides and the metal substrate and then diffuse away from the interface. In the case of immersion, the constant small adhesion reveals a stable surface charge with different periods of immersion time. We conclude that there is an equilibrium between the production of metal cations by dissolution and diffusion of the ions into the solution, which entails the constant surface charge. In other words, during immersion in phosphate buffer, the ion transfer is limited by the dissolution rate. Anodic polarization in phosphate buffer with its stronger metal ion dissolution leads to an accumulation of cations on the surface and, thus, to a higher surface charge.

In NaCl solution, adhesion increases with immersion time, indicating an increased surface charge. We conclude that dissolution of metal ions occurs faster than their diffusion into solution in NaCl solution, that is, the ion transfer is limited by diffusion. The lack of passivation is in agreement with reports about a decrease in corrosion resistance in NaCl solution with immersion time due to the development of defects in the surface film [40–41].

The increase in the friction coefficient of the outer layer indicates the growth of the outer layer with increasing immersion time. More material of possibly higher shear strength is in contact with the sliding tip, which experiences, thus, a higher plowing resistance. The outer layer grows by precipitation of metal hydrates, which are formed when dissolved metal ions diffuse towards the solution. This growth of the outer layer involves the three physicochemical processes depicted in Figure 5d, which have been invoked to explain the bilayer structure found after polarization [30–31,42]. Metal cations react with water, or anions present in the solution, and form hydrated oxides and hydroxides at the interface between the outer layer and solution. These hydrates diffuse into the bulk solution, or partially transform as precipitates into the outer layer. The latter process may be enhanced as a result of hydrate accumulation into supersaturation close to the surface. As the immersion continues, the friction coefficient of the outer layer increases sublinearly with immersion time in phosphate buffer (Figure 5c). The corrosion resistance of the oxide film in passivating solutions was reported to increase with time during the first stage and then remain almost constant after a longer immersion time [43–45]. We suggest a similar development for MGs in phosphate buffer, where the protective effect of the inner layer becomes stronger with the immersion time, the dissolution becomes slower, and the growth rate of the outer layer decreases.

In NaCl solution, the friction coefficient of the outer layer is constant at a value smaller than that of the outer layer in phosphate buffer after long immersion. During immersion, the outer layer does not grow significantly in NaCl solution, although the adhesion data indicated stronger dissolution than in phosphate buffer. It has been reported that phosphate ions interact strongly and promote the precipitation of dissolved metal hydrates due to the formation of insoluble metal phosphate species [28–29]. Such an accumulation of hydrates does not proceed in NaCl solution, and we conclude that the formation of metal hydrates is in equilibrium with their diffusion into the solution, or that the existing outer layer prevents the precipitation of further hydrates.

The friction coefficient for the outer layer after anodic polarization in phosphate buffer is much higher than that after immersion. During electrochemical polarization, a great quantity of dissolved ions diffuses as hydrates towards the solution in a short time, which become supersaturated near the metal surface and precipitate into the outer layer [30–31,42]. This supersaturation leads to the enhanced growth of the outer layer during polarization. When we combine these observations with those on adhesion, we conclude that the whole process depicted in Figure 5d is diffusion-controlled after polarization. Figure 5e summarizes how these physicochemical processes affect the friction and adhesion forces of the inner and outer layer.

We have attempted to determine the thickness of the outer layer by topographic measurements and by force–distance curves. While these methods clearly revealed a thickness of 0.2–0.3 nm for the outer layer after polarization [21], they did not show a clear distinction of the top of the outer layer after immersion. Maurice et al. [32] found that the thickness of the outer layers on stainless steel surfaces is similar during immersion and after polarization (0.5–0.7 nm) using XPS analysis. Taking into account the friction results reported here, we can only conclude that that the outer layer formed during immersion is less compact than that found after polarization with its stronger dissolution.

## Conclusion

Our results reveal the instructive connection between nanoscale friction and surface processes on a metallic glass upon immersion in corrosive solutions. Friction coefficients indicate the development of the passivated inner layer of the surface and the growth of a precipitated and displaceable outer layer. Adhesion indicates the accumulation of charge at their interface. The evolution of friction with increasing immersion time reveals the interrelation of relevant physicochemical processes, namely the production of metal cations by surface dissolution at the interfaces of two layers, the diffusion of ions to the interface of outer layer and solution, the formation of hydrates at the surface, and the competition between diffusion of hydrates into solution and their precipitation into a growing outer layer. Understanding the mechanisms of nanoscale friction on metallic glasses is a basis for applications involving mechanical contacts under corrosive conditions. Also, nanotribology offers unique methods to resolve the microscopic corrosion process in situ.

Although results were reported here for metallic glasses, we suggest that the study of surface layers and charges by nanotribology can be extended to the understanding of corrosion mechanisms in other metal and alloy systems. Future studies can exploit the lateral resolution of scanning force microscopy to detect dissolution and precipitation on selected areas of interest such as different phases, grains, and inclusions [46].

## Materials and Methods

Zr_63_Ni_22_Ti_15_ (ZrNiTi) MG ribbons were produced by the single-roller melt-spinning technique and provided by the Physics Institute at the University of Basel (Switzerland). The X-ray diffraction of Cu Kα radiation (XRD) verified the amorphous nature of the ribbons.

All friction experiments were conducted at room temperature in 0.2 M phosphate buffer (Na_2_HPO_4_ + NaH_2_PO_4_, pH ≈ 7) and 0.2 M NaCl solution. The original surfaces of the tested ribbons are flat with a surface roughness less than ca. 1 nm. Friction experiments were carried out after immersing a new sample into the solution for 1, 24, or 72 h. The exposed area of the samples was ca. 2.0 cm^2^ and ca. 1.0 mL of corrosive solution was added. For these experiments, we used an electrochemical atomic force microscope (ECAFM, Agilent 5500) and the oxidized tip (radius of ca. 30 nm) of a single-crystalline Si cantilever (PPP-CONT, NanoSensors, Germany). We adopted the beam geometry method to calibrate the force constants of the cantilever [47]. The resonance frequency of the cantilever at the first normal oscillation mode measured in the air was used to calculate the thickness of the cantilever [47]. The AFM tip sliding velocity was 8.0 μm·s^−1^ and the scan field was 1.0 × 0.125 μm^2^. Sixteen cycles of repetitive scans, each 64 scan lines, were performed in each scan field at a constant applied load and repeated on different surface areas with different loads, while the friction force was recorded. No wear of the AFM tip was observed by means of scanning electron microscopy (SEM) after selected friction measurements.

In order to establish differences in corrosion of ZrNiTi MGs between the solutions using a standard procedure, potentiodynamic polarization experiments were performed in the range of −0.5 to 1.5 V at a potential sweep rate of 1.0 mV·s^−1^, in a custom-made cell with a three-electrode setup. The MG ribbon, a miniature Ag/AgCl electrode, and a Au wire served as working, reference, and counter electrode, respectively. The polarization test was a separate experiment and subsequent friction experiments were performed using new samples, which were immersed without applying a potential.

## Supporting Information

Additional AFM measurements of repetitive scans with increasing normal loads are provided in Supporting Information File 1 to identify a possible influence of plastic deformation of the MG substrate. Decreasing friction force with repetitive scans was observed before in friction measurements on a graphene-coated Cu substrate [33]. In this work, when the applied load was increased stepwise, the friction force exhibited a sudden increase followed by a decay with scanning at all loads. This friction decay was attributed to the consecutive plastic deformation and hardening of the Cu substrate during repeated scanning. Different from strain hardening in conventional metals, MGs show strain softening induced by the creation of additional free volume during deformation [48–49]. No plastic strain occurs in this work, given the much higher yield strength of Zr-based MGs (approx. 1.7 GPa [50]) than that of copper (69–365 MPa [51]). The maximum contact pressure in this work is ca. 0.49 GPa (JKR model), smaller than the yield strength of MGs. As a contrast, we performed similar friction experiments. In NaCl solution, the friction decay can only be observed for the first applied normal load. With increasing the load, the friction force presents a sudden increase and then remains constant. The stable value of the friction force increases with the applied load. The friction coefficient is approx. 0.16 and the adhesion force is approx. 1.68 nN, similar to the values in Figure 5a. We conclude that the decreased friction in this work is not caused by the plastic deformation of the substrate but by the gradual removal of the outer layer. At the first load, the outer layer is completely removed from the surface. Thus, the tip just slides on the inner layer and shows a constant friction force in subsequent friction experiments at other loads.

File 1 Repetitive scans with increasing normal loads.
