# Supplementary material for: Relationship between corrosion and nanoscale friction on a metallic glass
Source: Beilstein J Nanotechnol. 2022 Feb 18;13:236–44. doi: 10.3762/bjnano.13.18 (PMC8895037; doi:10.3762/bjnano.13.18)
Supplement: File 1 — Repetitive scans with increasing normal loads. [file Beilstein_J_Nanotechnol-13-236-s001.pdf]

BEILSTEIN JOURNAL OF NANOTECHNOLOGY

Supporting Information

for

Relationship between corrosion and nanoscale friction on a metallic glass

Haoran Ma and Roland Bennewitz

Beilstein J. Nanotechnol. **2022,** *13,* 236–244. doi:10.3762/bjnano.13.18

Repetitive scans with increasing normal loads

License and Terms: This is a supporting information file under the terms of the Creative Commons Attribution License (https://creativecommons.org/licenses/by/4.0). Please note that the reuse, redistribution and reproduction in particular requires that the author(s) and source are credited and that individual graphics may be subject to special legal provisions.
The license is subject to the *Beilstein Journal of Nanotechnology* terms and conditions: (https://www.beilstein-journals.org/bjnano/terms)

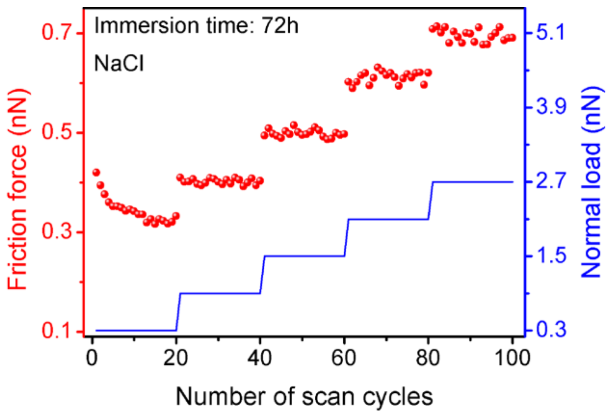

Figure S1: Friction force as a function of number of scan cycles on $Zr_{63}Ni_{22}Ti_{15}$ metallic glass after immersion into 0.2 M NaCl solution for 72 h, with stepwise increasing the normal load.
